# Detectability and impact of repetitive surveys on threatened West African crocodylians

**DOI:** 10.1002/ece3.8188

**Published:** 2021-10-07

**Authors:** Michel N. Ahizi, Christine Y. Kouman, Allassane Ouattara, N’Dri Pascal Kouamé, Azani Dede, Emilie Fairet, Matthew H. Shirley

**Affiliations:** ^1^ Université Nangui‐Abrogoua Abidjan Côte d'Ivoire; ^2^ Project Mecistops Sarasota Florida USA; ^3^ Office Ivoirien des Parcs et Réserves Abidjan Côte d'Ivoire; ^4^ Wildlife Conservation Society New York New York USA; ^5^ Institute of Environment Florida International University North Miami Florida USA; ^6^ Rare Species Conservatory Foundation Loxahatchee Florida USA

**Keywords:** *cataphractus*, Côte d’Ivoire, *Crocodylus*, detection probability, *Mecistops*, *niloticus*, *suchus*, wariness

## Abstract

West African crocodylians are among the most threatened and least studied crocodylian species globally. Assessing population status and establishing a basis for population monitoring is the highest priority action for this region. Monitoring of crocodiles is influenced by many factors that affect detectability, including environmental variables and individual‐ or population‐level wariness. We investigated how these factors affect detectability and counts of the critically endangered *Mecistops cataphractus* and the newly recognized *Crocodylus suchus*. We implemented 195 repetitive surveys at 38 sites across Côte d’Ivoire between 2014 and 2019. We used an occupancy‐based approach and a count‐based GLMM analysis to determine the effect of environmental and anthropogenic variables on detection and modeled crocodile wariness over repetitive surveys. Despite their rarity and level of threat, detection probability of both species was relatively high (0.75 for *M. cataphractus* and 0.81 for *C*. *suchus*), but a minimum of two surveys were required to infer absence of either species with 90% confidence. We found that detection of *M*. *cataphractus* was significantly negatively influenced by fishing net encounter rate, while high temperature for the previous 48 h of the day of the survey increased *C*. *suchus* detection. Precipitation and aquatic vegetation had significant negative and positive influence, respectively, on *M*. *cataphractus* counts and showed the opposite effect for *C*. *suchus* counts. We also found that fishing encounter rate had a significant negative effect on *C*. *suchus* counts. Interestingly, survey repetition did not generally affect wariness for either species, though there was some indication that at least *M*. *cataphractus* was more wary by the fourth replicate. These results are informative for designing future survey and monitoring protocols for these threatened crocodylians in West Africa and for other endangered crocodylians globally.

## Graphical abstract

Establishing population monitoring to assess West Africa crocodilian status is a priority action. Despite their high detectability, *Mecistops cataphractus* and *Crocodylus suchus* detection and counts were influenced by both environmental and anthropogenic factors. They also generally exhibited similar wariness response to repetitive surveys.

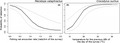

## 1 INTRODUCTION

Monitoring programs are crucial for informing biodiversity management (Jones et al., 2011), especially for species of conservation concern. But inference of species population trends can be strongly influenced by imperfect detection, especially for cryptic and secretive species (Bauder et al., 2017). Species detectability is central to appropriate survey design (MacKenzie et al., 2002), but can vary widely among even closely related species (Wintle et al., 2005) and be heavily influenced by environmental factors (Burns et al., 2019), habitat features, and observer bias (Jeffress et al., 2011; Shirley et al., 2012).

Crocodylians are difficult species to survey and monitor (Bayliss, 1987) because they are principally nocturnal, behaviorally cryptic, and surveys are impeded by significant submersion bias (i.e., only a fraction of the population is at the surface and detectable at any given time; Bugbee, 2008). Previous studies have shown that environmental variables, ranging from moon phase, water level, cloud cover, wind speed, wave height, mean water temperature, and maximum temperature the day of the survey (Da Silveira et al., 2008; Hutton & Woolhouse, 1989; Pacheco, 1996a; Woodward & Marion, 1978), as well as observer‐specific factors (Shirley et al., 2012), can have significant influence on crocodylian detectability (Table 1). For example, greater moonlight and higher water temperatures can increase detection, while high water levels and cloud cover can decrease detection (Bugbee, 2008). Bugbee (2008) and Carter (2010) showed that these environmental variables largely impact detection through increased or decreased emergence rates.

**TABLE 1:** Environmental and anthropogenic factors that have been shown to influence detectability of crocodylian species

	*Alligator mississippiensis* ^1/6^	*Crocodylus niloticus* ^2^	*Caiman latirostris* ^3^	*Melanosuchus niger* ^4/5^	*Caiman crocodilus* ^5^	*Mecistops cataphractus^7^*	*Crocodylus suchus^7^*
Precipitation	●/−	†	●	†/†	†	−	+
Water Level	−/−	−	†	†/−	−	†	†
Water Temperature	+/−	●	●	●/†	†	†	†
Presence/Absence Wind	†	●	†	†/†	†	†	†
Wind speed	†/−	†	●	−/†	†	●	●
Presence/Absence Waves	†	●	†	†/†	†	†	†
Wave Height	−		†	●/†	†	●	●
Moon Phase	+/+	●	●	●/+	●	●	●
Presence/Absence Moonlight	†	●	+	†/†	†	†	†
Cloud Cover	+	●	●	−/†	†	†	†
Mean Air Temperature	●/+	+	●	† /†	†	●	●
Max. Air Temp. 24 h Prior	●	●	†	† /†	†	†	†
Min. Air Temp. 24 h Prior	●	●	†	† /†	†	†	†
Difference between mean water and mean air temperature	†	+	†	† /†	†	†	†
Maximum air temperature the day	†	†	†	+/†	†	†	†
Fishing net encounter rate	†	†	†	†	†	−	−
Aquatic vegetation	†	†	†	†	†	+	−
Mean air temperature the previous 48 h	†	†	†	†	†	●	+
Mean daily precipitation the previous 48 h	†	†	†	†	†	−	●
Precipitation the day prior to the survey	†	†	†	†	†	●	●

Each of these factors had either no relationship (●), negative relationship (−), positive relationship (+), or was not tested (†). Sources: 1: Woodward and Marion (1978), 2: Hutton and Woolhouse (1989), 3: Sarkis‐Gonçalves et al. (2004), 4: Pacheco (1996b), 5: Da Silveira et al. (2008), 6: Bugbee (2008), 7: this study.

These previous studies, while comprehensive, are limited to a small number of species and mostly employed analytical methods that are under question, and thus, this issue should be revisited. For example, stepwise regression was a frequently employed analytical method thought useful for selecting explanatory variables in linear models, but has fallen under criticism (Derksen & Keselman, 1992; Weiss, 1995) because it suffers from a multiple testing problem and is biased (Mundry & Nunn, 2009). More recent efforts have utilized generalized linear mixed modeling (GLMM; Strickland et al., 2018) or occupancy models with occurrence data (Gardner et al. (2016), which overcome some of the issues with stepwise regression, to assess the impact of environmental variables on alligator counts and occupancy, respectively. Both studies, using these less controversial analytical methods, supported some previous findings that, for example, water temperature has a positive effect on crocodylian counts, while recent rainfall has a negative effect.

Virtually, all of these previous studies were conducted on *Alligator mississippiensis*, *Caiman crocodilus*, and *Crocodylus niloticus*—among the most abundant and well‐studied crocodylians in the world. The crocodylians of West Africa, on the contrary, are among the most threatened and least researched species in the world. The West African slender‐snouted crocodile (*Mecistops cataphractus*) is listed as Critically Endangered on the IUCN Red List (Shirley, 2014), and the highest priority action for this species is to assess population status throughout its range as a basis for establishing *in situ* conservation programs (Shirley, 2010). This species inhabits rivers mainly covered by dense or shady vegetation (Shirley et al., 2018). *Mecistops cataphractus* displays some tolerance of forest replacement by agriculture when human and fishing pressure are absent (Shirley et al., 2009). It is highly aquatic, depending on the rainy season and high water levels for all phases of the reproduction cycle (Shirley et al., 2018). In contrast, the West African crocodile (*Crocodylus suchus*) is much more widely distributed and generalist in its use of habitats (Luiselli et al., 2012). Previously thought to be just odd populations of the Nile crocodile (Hekkala et al., 2011; Schmitz et al., 2003), this little researched species meets the criteria for Endangered on the IUCN Red List (M.H. Shirley, unpub. data), and there is a great need for more surveys and ecological studies (Fergusson, 2010). Both species are faced with extreme habitat loss, overfishing, and the increasing threat of artisanal mining activities.

Crocodylian surveys already underestimate population abundance due to the cryptic nature and behavior of the members of this Order. These effects are exacerbated for the rare, shy, and overexploited species—like *M. cataphractus* and *C. suchus*—for which the risk and bias from imperfect detection has real impacts on decision‐making. For such elusive species, use of occupancy models based on occurrence data may be more appropriate than count data for monitoring programs (Ward et al., 2017). Occupancy methods that deal with imperfect detection are increasingly popular in the herpetological community (e.g., Chandler et al., 2020; Halstead et al., 2018; Petitot et al., 2014). Through data from repetitive surveys, occupancy models allow for the estimation of the probability of site occupancy and detection probability (Kery, 2002; Pellet & Schmidt, 2005). And, in fact, repetitive surveys are already regularly employed in crocodylian monitoring programs to overcome detection biases (Fukuda et al., 2013; Messel et al., 1981; Webb et al., 2000). However, it is not yet known whether repeat surveys over a relatively short period of time actually improve detectability of crocodylians or increase wariness, which could lead to decreases in detection probability (Fieberg et al., 2015) due to increased submergence and other fleeing/evasive behaviors. This is all further complicated by anthropogenic pressures on these species, which can drastically influence the behavior of crocodylians (Lang, 1987; Pacheco, 1996a).

We aimed to evaluate the factors influencing detection and wariness of *M. cataphractus* and *C. suchus*, two highly threatened West African crocodylians. To compare with previous studies using linear models and crocodylian counts, we implemented GLMM on our repetitive count data. However, due to the rarity of our studied species, we also evaluated these covariates in a single season occupancy modeling framework. We further sought to provide advice for future monitoring programs through an evaluation of the minimum survey effort needed to infer species absence and of how repetitive surveys impact wariness.

## 2 METHODS

### 2.1 Study sites

**FIGURE 1:**
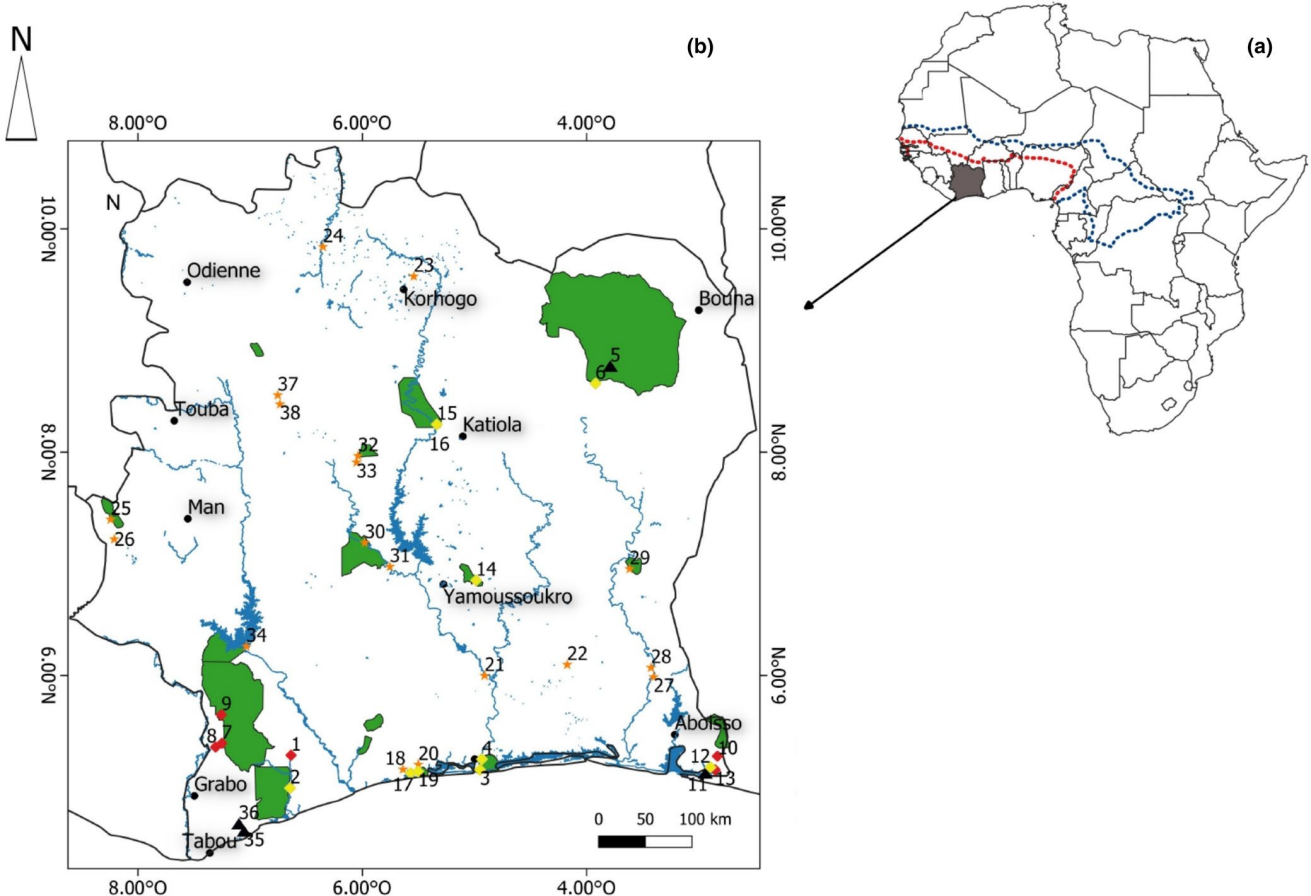
Global distribution (a; *Mecistops cataphractus* = red dotted line, *Crocodylus suchus* =blue dotted line) and location of surveys sites (b) for *M. cataphractus* and *C. suchus* in Côte d’Ivoire, West Africa. Symbology on the (b) map represents: sites where only *M. cataphractus* was observed (red diamonds), sites where only *C. suchus* was observed (yellow diamonds), sites where both species were observed (black triangles), sites where no crocodiles were observed (orange stars), and cities (black dots). Green shaded polygons are national parks and blue shading is water

From 2014 to 2019, we conducted surveys of *M. cataphractus* and *C. suchus* in different habitat types throughout Côte d’Ivoire. We sampled 38 sites across the major ecoregions of the country, which are representative of those of West Africa: Guinean forest (50% of the country), the Sudano‐Guinean zone (19% of the country), and the Sudanian region (31% of the country; Figure 1). The Guinean forest zone is situated in the south and is characterized by high rainfall (>1600 mm/year) and predominantly Upper Guinea forest habitat. The Sudano‐Guinean zone is the transition zone between forest and savannah (Lauginie, 2007). Both zones are characterized by four seasons—long dry (November–February), long rainy (March–June), short dry (July–August), and short rainy (September–October). The Sudanian zone is located in the north and is characterized as a savannah region with single rainy (May/June–November) and dry (December–May) seasons. Throughout the country, average temperatures range from 24 to 32℃ (Brou, 2005).

### 2.2 Crocodiles surveys

We surveyed crocodiles predominantly during the dry season to increase detection rates (Fukuda et al., 2013). We conducted standard nocturnal spotlight surveys (Chabreck, 1966) from an inflatable, outboard‐powered boat with 15 hp engine at a cruising speed of about 5.0–6.0 km/h, by inflatable kayak, and/or on foot. A single observer conducted all surveys, who located crocodiles by their eyeshine using either a 78 lumen LED headlamp (80% of observations) or a 550 lumen LED spotlight (20% of observations), depending on the habitat, and approached individuals as close as possible to visually determine species and demographic class (i.e., by total length, TL). However, in this study, we did not include size class in any analyses. We classified crocodiles that submerged before species and total length could be determined as eyes only (EO). We tracked all survey routes and took waypoints for each crocodile sighting using a handheld GPS. Using a standard occupancy design (wherein each of S sites is visited K times; Mackenzie & Royle, 2005), we surveyed each site on three (from 2014 to middle 2016) or five (end of 2016 to 2019) consecutive nights. We surveyed a minimum survey distance of 10 km at each site on each occasion.

### 2.3 Environmental variables

We examined the influence of 10 environmental and anthropogenic variables that were previously shown to have significant influence on crocodile detection probability and are relevant to our study species and habitat (Table 1). We measured six of these variables in the field before or during each survey: moon phase (0–4), wave height and wind speed (0–3), precipitation the day prior to the survey (0, 1), the amount of vegetation present along the shoreline and fishing net encounter rate. We used a binary index of low or high vegetation where low vegetation denotes a visible shoreline with little to no overhanging vegetation and high aquatic vegetation denotes a shoreline completely covered by overhanging or aquatic vegetation (Gardner et al., 2016). We counted the number of fishing nets seen on the survey as an index of the subsistence fishing threat (Shirley et al., 2009). We assessed mean night air temperature and mean daily precipitation both on the day of the survey and for the previous 48 h from remote sensed data accessed through MODIS (Wan et al., 2015) and CHIRPS (Funk et al., 2015), respectively. Despite that water temperature and water level are known factors influencing crocodylian detectability, we did not include them here because we surveyed sites predominantly during the dry season and over only 3–5 consecutive days per site, effectively resulting in no variation within sites and any between site variation largely being a question of occupancy and not detectability.

Prior to further analysis, we standardized all continuous covariates and tested for multicollinearity among independent variables using the VIF function in the R package *car* (Fox & Weisberg, 2019). We found evidence of collinearity for wind speed with wave height and mean night air temperature the day of the survey with mean night temperature for the previous 48 h. Wave height is often correlated with wind speed (Woodward & Marion, 1978) and generally not significant in the small river systems where we surveyed, so we removed wave height from subsequent models. Likewise, we retained mean night temperature for the previous 48 h over mean night air temperature the day of the survey because of its more significant individual effect in subsequent models (Couturier et al., 2013). We conducted all subsequent analyses for each species independently.

### 2.4 Influence of environmental and anthropogenic variables on detection probability

We assessed the influence of environmental and anthropogenic variables on crocodile detection probability using both an occupancy framework and with linear mixed models. For both model types, we included all data from all surveys in all years, though treated missing data for repetitions four and five in years 2014 to mid‐2016 differently. We categorized missing values as NA in occupancy models, but used imputation methods to infer missing values in GLMM analyses (see below; Nakagawa & Freckleton, 2008).

Within the occupancy framework, we used a single season occupancy model to estimate detection probabilities (*p*) (MacKenzie et al., 2006). To do this, we created a detection history (0 = nondetection, 1 = detection) for each site across all the survey repetitions. For this analysis, we hypothesized that the populations were closed during the survey period, no heterogeneity in detection occurred, and the detection process was independent at each site (MacKenzie et al., 2002). We used the method of “plausible combination” (Bromaghin et al., 2013) for model selection and covariate evaluation, which is increasingly recognized as a robust multistage strategy to assess the fit of single season occupancy models (Morin et al., 2020). To derive detection probability and better understand the influence of covariates on detection, we paired the most general submodel for occupancy (ψ) with all candidate submodels for detection probability (*p*) using the *dredge* function in the package MuMin (Barton, 2020) and returned the best model (i.e., ∆AIC threshold of 0) (Morin et al., 2020). Ultimately, we assessed all combinations of the best detection covariates. Because our focus was exclusively on detection probability, we held occupancy constant in the final analysis (i.e., (*ψ*.)p[covariate]) (Kroll et al., 2008; Moreira et al., 2016; Phumanee et al., 2020), a standard practice when focused on one component, or the other, in occupancy‐based analyses (Cook et al., 2011; Jeffress et al., 2011; Wagner et al., 2019). We ranked models using Akaike's information criterion corrected for small sample size (AICc) and considered all models with ∆AICc ≤ 2 to be competitive models (Burnham & Anderson, 2002). We considered a covariate significant if the 95% CI did not include zero (Bauder et al., 2017). We conducted all analyses using the packages unmarked (Fiske et al., 2017) and AlCcmodavg (Mazerolle, 2015) in R v4.0.2 (R Development Core Team, 2020).

For GLMM analysis, as our surveys varied from three to five replicates, we replaced missing values (8.57% of the total dataset) in all sites with less than five replicates using a multiple imputation procedure (Nakagawa & Freckleton, 2008). Specifically, we used multiple imputation to fill in 18 (of 210; 8.6%) missing values for each of crocodile encounter rate, moon phase, wind speed, amount of aquatic vegetation, fishing net encounter rate, and precipitation the day prior to the survey. We preferred multiple imputation (MI) over single imputation (e.g., substituting missing values with global means) because of the small sample size and risk of underestimating the errors (Nakagawa & Freckleton, 2008). Further, Nakagawa and Freckleton (2011) found that, with mixed linear models, replacing missing values through MI results in better estimates of Akaike weights and standard errors compared to leaving NAs in the dataset. We generated and combined 100 imputed datasets (Graham et al., 2007) using the R package mice (Buuren & Groothuis‐Oudshoorn, 2011). After imputation, we determined whether the MICE algorithm has converged by plotting parameters against the iteration number and found no definite trends, indicating good convergence in the dataset including imputed values (Buuren & Groothuis‐Oudshoorn, 2011). We modeled crocodile counts using the *lmer* function in the lme4 package (Bates et al., 2015). We tested the same eight covariates included in the occupancy analysis as fixed effects with site as a random effect. We fit 256 combinations for each species, including the null and global models, without interaction terms and ranked models by AICc. We considered all models with ∆AICc ≤ 2 to be competitive models (Burnham & Anderson, 2002). We obtained model‐averaged coefficients using the *Model*.*avg* function in MuMin. Model‐averaged coefficients offer more reliable and robust point and uncertainty estimations of parameters (Burnham & Anderson, 2002; Richards et al., 2011; Symonds & Moussalli, 2011). For each coefficient, we report associate 95% confidence intervals (CIs) and the coefficient estimate with shrinkage (also called “zero method”). We considered a covariate significant if the 95% CI did not include zero (Bauder et al., 2017).

### 2.5 Estimating the minimum number of visits to infer absence

We used the mean estimate of detection probability from significant covariates of the occupancy‐based models for each species to estimate the minimum number of repetitive surveys required to determine that the species is truly absent from a site with 90% (Sliwinski et al., 2016) and 95% confidence (Barata et al., 2017). We calculated *N*_min_ using the expression of Pellet and Schmidt (2005):

$$N_{\min}=\;\log\left(1\;‐\;\text{CL}\right)\;/\;\log\left(1\;‐\;p\right)$$

where *P* denotes the detection probability, and CL is the desired probability of detecting the species at an occupied site on at least one of the *N*_min_ repetitive surveys.

We also used an occupancy analysis to examine differences in detection probability over the survey replicates (e.g., from 1 to 5). To do this, we fit an occupancy model using the most significant factors influencing each species detection (i.e., fishing encounter rate for *M. cataphractus* and mean temperature for the previous 48 h for *C. suchus*; see results) and survey replicate number as detection covariates. We then predicted from this model the replicate‐specific detection probability for each species. We implemented this analysis in R using the package unmarked (Fiske et al., 2017).

### 2.6 Effect of repetitive surveys on crocodile wariness

We used proportion of “EO” and zero detections across repeated surveys as indexes to examine the effect of repetitive surveys on crocodile wariness. We considered that a progressive reduction in the number of individuals seen or formally identified (i.e., increase in the number of “EO”) during surveys to be a reflection of an increase in wariness. For each site where the presence of each crocodile species was confirmed, we determined the proportion of observations that were EO (EO/number of all observations) for each survey, where 0.0 represented no wariness and 1.0 (represented by either 100% EO observations or zero detections) represented complete wariness. For sites where the two studied species were sympatric, we partitioned the EO observations by the ratio of the number of individuals actually attributable to either species.

Because our surveys varied from three to five replicates, we used multiple imputation as described above to estimate missing values for the following covariates: index of wariness, aquatic vegetation, fishing net encounter rate, and crocodile abundance for both species, resulting in 10.9% and 7.14% values derived from MI for *M. cataphractus* and *C. suchus*, respectively. We assessed crocodile wariness as a function of the survey replicate, the most important covariates for each species identified in both occupancy and count‐based GLMM analyses above, and crocodile encounter rate as an index of abundance. We included encounter rate because we hypothesized that population abundance may represent unmeasured disturbance effects on individual wariness (i.e., for rare species, higher abundance sites likely have less impacted or threatened population histories, which may capture unmeasured/unmeasurable histories of disturbance or harassment of individuals). The *M. cataphractus* model included survey repetition, abundance, fishing net encounter rate, precipitation the day of the survey and for the previous 48 h, and aquatic vegetation as fixed factors. The model for *C. suchus* included survey repetition, abundance, fishing net encounter rate, precipitation the day of the survey, temperature for the previous 48 h of the day of the survey, and aquatic vegetation as fixed factors. Both species models included site as a random effect. We used generalized linear mixed effects modeling, fit by restricted maximum likelihood, using the package *lme4* (Bates et al., 2015) and Satterthawaite's approximation from package *lmerTest* (Kuznetsova et al., 2017) to assess each factor's significance in R v4.0.2 (R Development Core Team, 2020).

## 3 RESULTS

### 3.1 Crocodile surveys and environmental variables

We conducted 195 surveys over 38 sites. We encountered only *M. cataphractus* at six sites, only *C. suchus* at nine sites, both species at four sites, and no crocodiles at 18 sites. Mean night air temperature the day of the survey was 21.92℃ (range 19.69–26.17℃) and 21.90℃ (range 19.45–25.8℃) for the previous 48 h. Precipitation varied from 0 to 2.8 cm (mean 0.1 ± 0.5) the day of the survey and from 0 to 5.1 cm (mean 0.4 ± 0.8) for the previous 48 h. The mean fishing net encounter rate was 0.65 ± 0.75 nets/km (range 0–2.75). The mode of moon phase (no moon) and wind were both 0, and aquatic vegetation was generally low across all sites.

### 3.2 Environmental variables influencing detection

**FIGURE 2:**
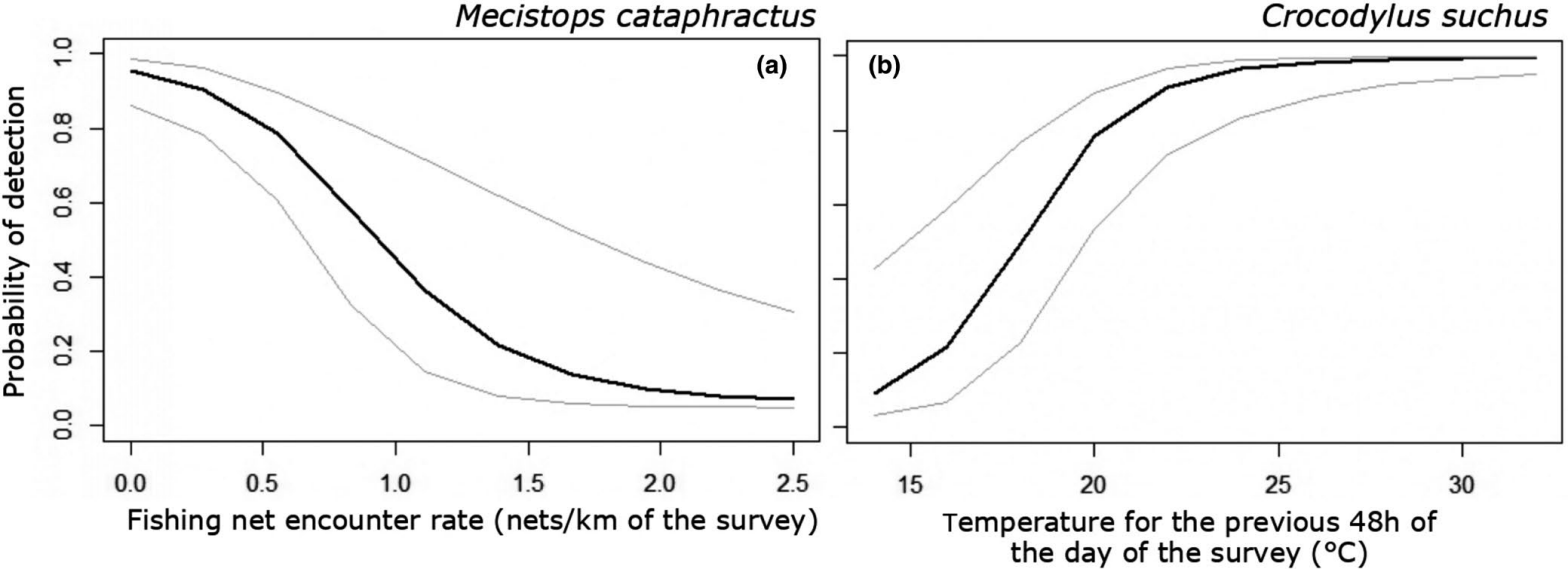
Relationship between (a) *Mecistops cataphractus* and (b) *Crocodylus suchus* detection probability and significant covariates identified from the best supported (AICc ≤ 2) occupancy model. In both tiles, solid lines represent the model‐averaged predicted values and dotted lines indicate the 95% credible intervals

Detection probability for *M. cataphractus* ranged from 0.75 to 0.87 and for *C. suchus* from 0.76 to 0.9 across survey sites and replicates. From our multistage model selection of occupancy‐based models, we found that two models for *M. cataphractus* (weight = 0.9) and four models for *C. suchus* (weight = 0.91) had ∆AICc ≤ 2 (Table 2). We found a significant negative correlation between *M. cataphractus* detection and fishing net encounter rate (Figure 2A) (−3.12, 95% CI: −5.1 to −1.1), as well as a nonsignificant relationship with moon phase (−0.56, 95% CI: −1.3 to 0.18) (Table 3). *Crocodylus suchus* detection was significantly positively correlated with mean temperature for the previous 48 h of the day of the survey (Figure 2B) (0.49, 95% CI: 0.16 to 0.81), while moon phase (0.42 95% CI: −0.13 to 0.97) and wind (1.39, 95% CI: −0.31 to 3.09) were not significant (Table 3).

**TABLE 2:** Best supported models (ΔAICc ≤ 2) for occupancy and counts (GLMM) of *Mecistops cataphractus* and *Crocodylus suchus*

Approach	Model structure	*K*	AICc	ΔAICc	Weight
*Mecistops cataphractus*					
Occupancy	Ψ (.) p(fishing net)	3	93.21	0.00	0.46
Occupancy	Ψ (.) p(fishing net + moon phase)	4	93.28	0.06	0.44
*Crocodylus suchus*					
Occupancy	Ψ (.) p(T_48 h + wind + moon)	5	134.19	0.00	0.30
Occupancy	Ψ (.) p(T_48 h + wind)	4	134.65	0.45	0.24
Occupancy	Ψ (.) p(T_48 h)	3	134.99	0.79	0.20
Occupancy	Ψ (.) p(T_48 h + moon)	4	135.28	1.08	0.17
*Mecistops cataphractus*					
GLMM	Aquatic + P_day + P_48 + fish	7	149.34	0.00	0.33
GLMM	Aquatic + P_day + P_48 + fish + moon	8	150.18	0.83	0.22
GLMM	Aquatic + P_day + P_48	6	150.39	1.04	0.20
GLMM	Aquatic + P_day + P_48 + fish + moon + prior	8	151.23	1.89	0.13
GLMM	Aquatic + P_day + P_48 + fish + moon + T_48	8	151.31	1.97	0.12
*Crocodylus suchus*					
GLMM	Aquatic + fish + P_day + T_48	7	−265.96	0.00	0.32
GLMM	Aquatic + fish + P_day	6	−264.62	1.33	0.16
GLMM	Aquatic + fish + P_day + T_48 + wind	8	−264.40	1.56	0.15
GLMM	Aquatic + fish + P_day + T_48 + prior	8	−264.22	1.73	0.13
GLMM	Aquatic + fish + P_day + T_48 + P_48	8	−264.03	1.93	0.12
GLMM	Aquatic + fish + T_48	6	−264.01	1.94	0.12

In the models, fish/fishing net = fishing net encounter rate (nets/km), moon = moon phase, P_48 = precipitation for the previous 48 h of the day of the survey, prior = precipitation the day prior to the survey, P_day = precipitation the day of the survey, T_48 = temperature for the previous 48 h of the day of the survey, aquatic = aquatic vegetation, and wind = wind speed, which were all included as fixed effects. *K* is the number of parameters, AICc: Akaike information criterion corrected for small sample, ΔAICc is the different from the best model’s AICc, and Weight is the Akaike weight of the model.

**TABLE 3:** Relative strength of association between detection of *Mecistops cataphractus* and *Crocodylus suchus* and each covariate included in the highest ranked occupancy models for each species. T_48 h = temperature for the previous 48 h of the day of the survey

Covariates	# of Models included	Model‐averaged estimate (95% CI)
*Mecistops cataphractus*		
Fishing net encounter rate (nets/km)	2	−3.12 (−5.10 to −1.15)
Moon phase	2	−0.56 (−1.30 to 0.18)
*Crocodylus suchus*		
Moon phase	4	0.42 (−0.13 to 0.97)
Wind	4	1.39 (−0.31 to 3.09)
T_48 h	4	0.49 (0.16 to 0.81)

The GLMM analysis revealed that six candidate models for *M. cataphractus* detection had strong support (∆AICc < 2) (Table 2). All the best models included aquatic vegetation, precipitation the day of the survey, and precipitation the previous 48 h (Table 2). Precipitation had significant negative effect on *M. cataphractus* counts, while aquatic vegetation had a significant positive effect (Table 4). Fishing net encounter rate, precipitation the day prior to the survey, moon phase, and mean air temperature for the previous 48 h of the survey had nonsignificant effects on *M. cataphractus* counts (Table 4).

**TABLE 4:** Coefficients from each covariate identified as having an impact on *Mecistops cataphractus* and *Crocodylus suchus* counts through GLMM

Parameters	Estimate	SE	Estimate (shrinkage)	LCI	UCI
*Mecistops cataphractus*					
Intercept	0.06	0.06	0.06	−0.06	0.18
Fishing	−0.06	0.03	−0.04	−0.13	0.00
**P_48**	**−0.06**	**0.02**	**−0.05**	**−0.11**	**−0.01**
**P_day**	**−0.08**	**0.02**	**−0.07**	**−0.12**	**−0.03**
**Aquatic vegetation**	**0.19**	**0.08**	**0.17**	**0.04**	**0.34**
Moon phase	0.03	0.03	0.01	−0.02	0.08
Prior	0.03	0.05	0.01	−0.07	0.12
T_48	0.02	0.04	0.01	−0.06	0.09
*Crocodylus suchus*					
(Intercept)	0.11	0.03	0.11	0.06	0.16
**Fishing**	**−0.03**	**0.01**	**−0.03**	**−0.06**	**−0.01**
**P_day**	**0.02**	**0.01**	**0.01**	**0.00**	**0.03**
T_48	0.03	0.02	0.02	−0.01	0.06
**Aquatic vegetation**	**−0.12**	**0.03**	**−0.12**	**−0.18**	**−0.06**
Wind	−0.02	0.03	0.01	−0.08	0.04
Prior	0.01	0.02	0.00	−0.02	0.04
P_48	0.00	0.01	0.00	−0.01	0.02

Illustrated models are the top models only. Fishing = fishing net encounter rate (nets/km), P_48 = precipitation for the previous 48 h of the day of the survey, Prior = precipitation the day prior to the survey, P_Day = precipitation the day of the survey, T_48 = average temperature for the previous 48 h of the day of the survey, aquatic vegetation, wind, and moon phase were all included as fixed effects. LCI: Lower confidence interval, UCI: upper confidence interval. Estimate (shrinkage) refers to the estimated coefficient with shrinkage (also called “zero method”). Covariates in bold are those that had significant effect.

Six of 256 candidate models had strong support (∆AICc < 2) for *C. suchus* counts and all included aquatic vegetation, fishing net encounter rate, and precipitation the day of the survey (Table 2). The model averaged beta estimates showed a significant negative effect of aquatic vegetation and fishing net encounter rate, while precipitation the day of the survey had a significant positive effect on *C. suchus* counts (Table 4). Precipitation the day prior to the survey, precipitation and temperature for the previous 48 h of the day of the survey, and wind speed had no significant effect on counts of this species.

### 3.3 Minimum number of visits for confident detection

In the field, we detected *M. cataphractus* at 83.3% of sites during the first survey replicate and at 100% of sites where they occurred by the second survey replicate. *Crocodylus suchus* was detected at 86.6% of sites during the first survey replicate, at 93.3% by the second and third survey replicate, and at 100% of sites where they occurred by the fourth survey replicate. Based on significant covariates from the occupancy models, detection probability for *M. cataphractus* was 0.75 (estimated with the mean value for each covariate), requiring a minimum of two visits to have 90% confidence and three visits to have 95% confidence that this species was not present at a site. The estimated mean detection probability for *C. suchus* was 0.81, and a minimum of two visits are required to conclude with 90% and 95% confidence that the species was absent. We found a weak decrease in probability of detection from the first to the fifth replicate—from 0.80 to 0.67 for *M. cataphractus* and from 0.85 to 0.77 for *C. suchus* (Table 5).

**TABLE 5:** The impact of repetitive surveys and environmental factors on *Mecistops cataphractus* and *Crocodylus suchus* wariness

Effects	Parameters	*Mecistops cataphractus*				*Crocodylus suchus*			
		Estimate (SE)	T‐value	*p*‐value	Detection probability	Estimate (SE)	T‐value	*p*‐value	Detection probability
Random effect	Site	0.06 (0.24)		.**003**		0.04 (0.21)		.**04**	
Fixed effect	Survey 1	0.27 (0.14)	1.98	.06	.80	1.93 (1.01)	1.92	.06	.85
	Survey 2	0.21 (0.13)	1.55	.13	.77	0.06 (0.15)	0.41	.69	.83
	Survey 3	−0.01 (0.14)	−0.08	.93	.74	−0.08 (0.16)	−0.50	.62	.81
	**Survey 4**	**0.30 (0.14)**	**2.18**	.**04**	.71	0.02 (0.15)	0.14	.89	.79
	Survey 5	0.19 (0.14)	1.33	.19	.67	0.15 (0.15)	1.00	.32	.77
	Abundance	0.07 (0.11)	0.64	.52	–	0.25 (0.25)	0.99	.33	–
	P_48	−0.09 (0.06)	−1.68	.10	–	–	–	–	–
	Aquatic	−0.19 (0.14)	−1.35	.19		0.19 (0.16)	1.24	.22	–
	P_day	−0.07 (0.05)	−1.38	.17	–	−0.05 (0.05)	−0.88	.38	–
	Fishing	0.10 (0.07)	1.43	.16	–	0.13 (0.14)	0.92	.36	–
	T_48 h	–	–	–	–	−0.08 (0.05)	−1.75	.09	–

The parameter estimates from GLMM‐based analysis and the replicate specific detection probability from occupancy analysis. Abundance = crocodile encounter rate (ind./km), Fishing = fishing encounter rate, Aquatic = aquatic vegetation, P_48 = precipitation for the previous 48 h of the survey, P_day = Precipitation the day of the survey, T_48 = average temperature for the previous 48 h of the day of the survey. (–) indicates that the parameter was not included in the model for that species. Fixed factors in bold indicate those that had a significant effect (*p *< .05).

### 3.4 Effect of repetitive surveys on crocodile wariness

Globally, we found no effect of survey repetition on wariness for either species, except for *M. cataphractus*, which showed an increase in wariness for replicate four (Table 5). None of environmental and anthropogenic factors nor abundance had significant effect on wariness of both species.

## 4 DISCUSSION

Understanding species detectability and identifying environmental factors that influence species detection and, therefore, abundance or occupancy estimation are central to wildlife monitoring. Using both occupancy and count‐based approaches, we found that the environmental variables that best predict crocodylian species detection probability and count variability differ substantially both as a result of the analytical approach itself and between species.

Despite their considerably more threatened status, we found higher detectability for *M. cataphractus* and *C. suchus* (0.75 and 0.81, respectively) than has been reported previously for abundant crocodylians like the American alligator (0.16–0.62; Gardner et al., 2016). Though in both studies, crocodiles were surveyed during the best period for detection (June for Gardner et al., 2016 and dry season in our study), the fact that Gardner et al., 2016’s study took place in the extreme limit of American alligator distribution, while ours was in the core distribution of our study species, could at least partially explain the observed differences. We also mostly detected juveniles and subadults (80% <1.5m TL), which typically have higher detection rates and, for threatened crocodylians, are often the only individuals encountered to indicate species presence as adults can be incredibly wary.

In terms of factors affecting detectability of *M. cataphractus*, we found that fishing net encounter rate had a significant negative relationship with encounters. This study is the first to quantify the effect of fishing activities on crocodylian detection and counts. Overexploitation of freshwater fisheries is among the major threats to crocodiles in West Africa (Shirley et al., 2009). *Mecistops cataphractus*, in particular, is highly impacted by fishing because its longirostrine snout and tooth morphology drive a largely piscivorous prey base (Erickson et al., 2012), and they often drown in fishing nets (Shirley et al., 2018). Our results showed that precipitation the day of the survey and for the 48 h prior to the survey had significant negative effects on *M. cataphractus* counts. Precipitation is generally a key factor affecting ectotherm activity (Rozen‐Rechels et al., 2019) and, in other crocodylians, has been shown to increase submergence behavior (Bugbee, 2008). For forest species, like *Mecistops* spp. and likely *Tomistoma*, *Osteolaemus* spp., and *Paleosuchus* spp., precipitation likely amplifies the strong cooling effect already present in forested areas (Li et al., 2015), creating unfavorable microclimatic conditions for ectotherm thermoregulation (Falcón et al., 2018; Seebacher & Franklin, 2005). Though some forested crocodylian species use climbing behavior to increase opportunities for thermoregulation (e.g., Dinets et al., 2014), *M. cataphractus* has not yet been observed employing this behavior, suggesting it may be more susceptible than other, ecologically similar species to postprecipitation cooling.

Contrary to previous findings indicating that aquatic vegetation negatively affects crocodylian detection (e.g., Cherkiss et al., 2006), we found that *M. cataphractus* detection increases with increased aquatic vegetation. We observed that shorelines of forested rivers (where most *M. cataphractus* were encountered) were extensively covered by overhanging tree limbs and stilt roots. *Mecistops cataphractus* uses these structures as both refuge sites and for feeding due to abundance of prey that also congregate around the submerged structures (C. Kouman, pers. comm., 2021), resulting in them being more detectable because the stilt roots do not reduce observer visibility and, instead, likely provide indicators for where to search for the species. This is in contract to larger rivers, which were often more choked by invasive species like water hyacinth (*Eichhornia crassipes*), but which do not generally provide favorable habitat for *M. cataphractus*.

We recorded a significant positive effect of temperature for the previous 48 h of the survey on *C. suchus* detection. We surveyed crocodiles predominantly in the dry season, which is characterized by warmer temperatures during the day but cooler during the night—meaning that individuals likely take advantage of the increased thermoregulatory opportunities on hotter days. Additionally, we mostly observed small individuals (TL < 1.5 m), which have been shown to be relatively more active during lower temperature periods in other crocodylians (e.g., *Melanosuchus niger*; (Pacheco, 1996a). It is interesting to see that even small temperature fluctuations influence behavior change in more stable/less heterogeneous thermal environments, like the tropics. Even more interesting is the lack of significant effect on *M. cataphractus* detection and counts. Slender‐snouted crocodiles are predominantly associated with dense forested habitats, and though members of the genus are documented using fallen trees and such to bask in the absence of exposed banks (Dinets et al., 2014; Shirley et al., 2018), they are not typically associated with basking behavior like *Crocodylus* species. Further understanding of the thermoregulatory requirements of tropical, forest‐dwelling crocodylian species is necessary. Similar to previous studies (e.g., Cherkiss et al., 2006), but in contrast to what we found for *M. cataphractus*, aquatic vegetation had a significant negative influence on *C. suchus* counts. In large open rivers where we mostly found *C. suchus*, water hyacinth formed a compact floating vegetation mat along the riverbank that physically limits the ability of observers to see the crocodiles and may result in 50% or more of individuals going undetected (e.g., Thorbjarnarson, 1988). Surprisingly, we found that precipitation the day of the survey had significant positive effect on *C. suchus* counts. We did not survey during active rainfall, though surveyed on several occasions after daytime precipitation. Some studies have suggested that invertebrates, and fish and aquatic birds as a result, are more active after rains (e.g., Williams, 1951), perhaps driving increased activity levels in *C. suchus* as they forage. Like for *M. cataphractus*, *C. suchus* counts were significantly negatively affected by fishing nets. Regardless of species, overfishing is a major threat for aquatic species throughout West Africa. Interestingly, we also found a positive correlation between moon phase and fishing net encounter rate (*F *= 3.821, *df* = 4, *p *= .005), suggesting that increased fishing efforts with increasing moonlight could be an important factor driving submergence and detectability of crocodiles in heavily fished areas.

It should be noted that previous studies have identified water level as an important factor affecting crocodile detection and counts (Da Silveira et al., 2008; Hutton & Woolhouse, 1989). We did not account for this environmental variable in our work because we implemented all of our surveys in the dry season and did not observe water level heterogeneity across survey replicated. Future surveys that conducted either across seasons, or with repetitive survey timing that provides for heterogeneity in water level at sites could implement multiseason occupancy modeling to estimate both detection probability and evaluate how seasonal variation of environmental factors can influence it (Burns et al., 2019; Fujisaki et al., 2011).

Despite these factors impeding observation of these two crocodylian species in the field, and because of the relatively high detectability of both species, we estimated that relatively few visits (*n *= 3) are necessary to infer that either is absent at a given site with 95% confidence. This is confirmed by our field observation showing that, generally, we detected with 100% confidence either species after two surveys replicate. Even though *C. suchus* had greater detection probability, they were detected with 100% confidence at sites where they occurred by the fourth survey replicate. This was mostly driven by a drastic change in water level during our third survey at the Fresco lagoon, where the lagoon mouth was opened leading to a considerable decrease in water level and resulting in higher than expected detectability for replicates four and five. However, on average, we detected with 100% confidence either species after two surveys replicate. These results likely mean that we can conclude the true absence of *M. cataphractus* and *C. suchus* at all sites where we did not detect them—53% of our study sites. Another point which could reinforce this inference is the fact that we failed to detect any crocodiles in areas where fishing encounter rate was 2/km—further reinforcing the idea that these species are highly threatened by anthropogenic activities. However, because detection probability can be influenced by factors ranging from season (MacKenzie et al., 2003) to true population size (Tanadini & Schmidt, 2011) and survey effort (Jeffress et al., 2011), which were mostly unaccounted for in this work, this result should be interpreted with preliminary caution.

Our presence–absence versus count‐based analyses revealed how analytical approach can influence our understanding of how some environmental factors influence species detection and counts. Occupancy analysis, which addresses imperfect species detection, has garnered much attention because it allows for reliable and cost‐effective analysis of population distribution, habitat use, and relative abundance, with less demanding data inputs compared to abundance estimation approaches (Casner et al., 2014; Noon et al., 2012). While not explicitly accounting for detectability, generalized linear models (GLMs) and generalized linear mixed models (GLMMs) are also widely used due to their ease of application and ability to incorporate count data (Gorosito et al., 2018). For count data, Royle (2004) developed the N‐mixture model to provide reliable estimates of abundance, which incorporates imperfect detection. However, there have been cases illustrating poor fit of these models, especially to species with considerable detectability issues (e.g., Ward et al., 2017). In our case, our results showed that occupancy‐based detection and GLMM based on counts are driven by different predictors suggesting they can be complementary, as has been seen in other studies (Gorosito et al., 2018). We suggest researchers and managers consider results from multiple modeling approaches whenever possible to obtain a complete picture of species ecology to implement effective management strategies.

Though repetitive surveys are frequently employed to survey crocodylians, there has as yet been no assessment of crocodylian response to repetitive surveys. Using proportion of EO observations as an index of wariness, we found that both West African crocodylian species generally exhibited no wariness response to repetitive surveys, which is also confirmed by the only weak decrease in detection probability from repetition one to five. Previous research has assessed wariness in crocodile populations and found this type of indicator to reliably represent wariness (Pacheco, 1996b; Webb & Messel, 1979). Despite wariness can be influenced by a multitude of factors, including hunting pressure, habitat type (Punzo, 2007), and environmental (Van Dongen et al., 2015), and can also vary between populations and species. In the present study, we found that no environmental variables affected wariness of either species. This is similar to previous findings for other crocodylians (Pacheco, 1996b; Webb & Messel, 1979) and suggests that wariness is largely driven by interactions with people. Similarity of wariness response could be attributable to hunting pressure because crocodile hunting in West Africa is typically not species‐specific, at least not between *M. cataphractus* and *C. suchus*, as the primary objective of the hunters is simply acquisition of whatever is available. In addition, hunting pressure in West Africa occurred in all our study sites, even in protected areas, and is exacerbated in aquatic environments due to the presence of both fishermen and poachers (Ferreguetti et al., 2018) and the relatively little attention paid to aquatic environments by anti‐poaching teams. In spite of that, our models not find significant effect of fishing net encounter rate on crocodile wariness, suggesting that some other fishing/hunting related variable may be a better predictor of crocodylian wariness. Further research using another index of wariness, such as stress hormones, and other anthropogenic factors as covariates should be explored.

### 4.1 Implications for conservation

This study is the first to assess the effects of anthropogenic and environmental factors on *M. cataphractus* and *C. suchus*, two of the least known crocodylians worldwide, for management purposes. Information obtained will enhance our understanding of the ecology of these species and help to develop strategies for their monitoring and conservation. In order to increase detection and counts, we recommend that crocodylian surveys are planned at a time that is optimal for detection (i.e., dry season; Fukuda et al., 2013), but will not be confounded by abundant aquatic vegetation, like water hyacinth. Further, to optimize monitoring programs, a trade‐off between the number of sampling occasions (number of visits) and the number of sites must be met. In general, fewer sites require more sampling occasions to increase precision on the total population (Mackenzie & Royle, 2005); however, the minimum number of sampling occasions must reliably predict species absence (Barata et al., 2017). We found that three surveys were necessary to infer species absence which, in light of decreasing detection probability to survey replicate five and increase in wariness associated with survey four, likely means that three visits for any given site is sufficient to obtain reliable information. Limiting surveys to three replicates can reduce the costs of the monitoring program and allow time and finances for surveys of additional sites. Additionally, though there will be temptation to plan to surveys for sympatrically distributed species simultaneously, the reality is that environmental variables like precipitation and temperature may differentially impact species detectability. At the very least, this must be accounted for during data analysis, but ideally monitoring will be designed to optimize detection of each target species. In West Africa, the unprecedented deforestation rate is seemingly increasing the availability of habitat for *C. suchus*, resulting in increasing contact, and potentially competition, with *M. cataphractus*. The use of multispecies occupancy models accounting for the biotic and abiotic factors that influence detection and occupancy of these two species may be useful in improving monitoring‐based management decision‐making for these two species (Devarajan et al., 2020). Our results additionally support that, beyond the influence of environmental factors on species detection and counts, crocodylian researchers need to account for anthropogenic factors which could have profound impacts on species behavior and habitat use. In West Africa, and elsewhere, where crocodylian monitoring programs are increasingly established, accounting for these factors both in the field and through use of appropriate analytical approaches, will provide reliable information on population status and trends without being financially overburdensome. Finally, our result suggests that the likelihood of encountering viable crocodile populations decreases with increasing fishing pressure. Overexploitation of fish in inland waterways is an increasingly recognized global problem (Allan et al., 2005). It is likely that sustainable inland fisheries management will be critical to successful crocodile conservation in regions like West Africa moving forward.

## CONFLICT OF INTEREST

None declared.

## AUTHOR CONTRIBUTION

**Michel N. Ahizi:** Conceptualization (lead); Data curation (lead); Formal analysis (equal); Investigation (equal); Methodology (lead); Software (lead); Writing‐original draft (lead); Writing‐review & editing (equal). **Christine Y. Kouman:** Conceptualization (supporting); Data curation (equal); Formal analysis (supporting); Investigation (equal); Methodology (supporting); Software (supporting); Writing‐original draft (supporting); Writing‐review & editing (equal). **Allassane Ouattara:** Supervision (equal); Validation (equal); Visualization (equal); Writing‐review & editing (equal). **N'Dri Pascal Kouame:** Formal analysis (equal); Software (supporting); Validation (equal); Writing‐review & editing (equal). **Azani Dede:** Data curation (equal); Formal analysis (equal); Investigation (equal); Writing‐review & editing (equal). **Emilie Fairet:** Data curation (equal); Formal analysis (equal); Validation (equal); Writing‐review & editing (equal). **Matthew H. Shirley:** Conceptualization (supporting); Data curation (equal); Formal analysis (equal); Funding acquisition (lead); Investigation (equal); Methodology (supporting); Project administration (equal); Supervision (equal); Validation (equal); Writing‐original draft (equal); Writing‐review & editing (equal).

## Data Availability

Data used to generate the results and figures are available from the Dryad Digital Repository: https://doi.org/10.5061/dryad.15dv41nz5.
